# Periodontal Disease in Patients Visiting a Tertiary Care Dental Hospital: A Descriptive Cross-sectional Study

**DOI:** 10.31729/jnma.6463

**Published:** 2021-04-30

**Authors:** Bhageshwar Dhami, Kamal Babu Thapaliya, Dinesh Kumar Shrestha, Bidhan Bhandari, Sujaya Gupta

**Affiliations:** 1 Department of Periodontics, Kantipur Dental College, Kathmandu, Nepal; 2 Thapaliya Dental Clinic, Chitwan, Nepal; 3 Department of Dentistry, Kanti Children's Hospital, Kathmandu, Nepal; 4 Shree Nobel Dental Home, Kathmandu, Nepal; 5 Department of Periodontics and Oral Implantology, Kathmandu Medical College, Bhaktapur, Nepal

**Keywords:** *body mass index*, *community periodontal index*, *loss of attachment*, *oral hygiene index*, *smoking*

## Abstract

**Introduction:**

Periodontitis is chronic disease leading to tooth loss. Oral hygiene practices combined with regular dental examinations keep oral cavity disease free and maintain periodontal health. The primary objective was to find out the prevalence of periodontal disease of patients measured by the Simplified Oral Hygiene Index and Community Periodontal Index.

**Methods:**

This descriptive cross-sectional study was conducted in department of Periodontics of a tertiary care dental hospital from April to June 2019 after obtaining ethical clearance and informed consent. Participants were recruited by convenience sampling and 183 sample size was calculated. Proforma included demographics, Simplified Oral Hygiene Index, Community Periodontal Index, body mass index, and smoking status. Data were entered in Statistical Package for Social Sciences version 23 and descriptive statistics were presented as frequency, percentage, mean, and standard deviation.

**Results:**

Prevalence of periodontal disease corresponding to loss of attachment 1, 2, 3, and 4 was found to in 104 (56.83%) participants. Simplified Oral Hygiene Index score was 1.67±0.89 with “fair” status in majority 114 (62.30%). Gingivitis (Community Periodontal Index 1, 2) was seen in 136 (74.32%). The mean age was 36.37±14.43 years of which 92 (50.27%) were female but smoking was more in males.

**Conclusions:**

This study suggests deteriorating periodontal health related to age, sex, oral hygiene, smoking, and BMI. As updated information on oral and periodontal health in Nepal is limited, this assessment would help the national policy makers on oral health intervention measures to prevent periodontitis and develop future programs to improve oral health.

## INTRODUCTION

Oral health related quality of life is associated with periodontal status.^1^ Periodontal disease is chronic disease leading to tooth loss.^2–5^ Oral hygiene practices of regular brushing and flossing of teeth combined with regular dental examinations keep oral cavity healthy. Oral hygiene can be assessed by accumulation of soft and hard deposits on teeth surfaces, the aetiological factors of periodontal diseases.^6^

Using standard tools to assess the oral hygiene status and practices that affect the periodontal health status is essential in formulating appropriate and acceptable dental health services and oral health awareness programs to improve the oral health of the population.^2,3, 7^

The primary objective of the current study was to find the prevalence of periodontal disease of the participants and secondarily to observe the various factors affecting periodontal condition like age, gender, body mass index (BMI), and oral hygiene methods used by the individuals to maintain their periodontal health.

## METHODS

This single-centre descriptive cross-sectional study was conducted in the Department of Periodontics, Kantipur Dental College from April to June 2019 after obtaining the ethical clearance from the Institutional Review Committee (Ref. 3/2019). Informed consent was obtained before conducting interview and oral examination. The inclusion criteria were male and female individuals over 15 years of age with two or more teeth present in a sextant that were not indicated for extraction. Natural teeth with full coronal restorations and the teeth reduced in height by caries or trauma were excluded. Participants were recruited by convenience (non-probability) sampling and 183 sample size was calculated using formula:

$$\begin{array}{ll} \text{n} & \text{= }{\text{Z}}^{\text{2}}\times \text{p}\times (1-\text{p)}/{\text{e}}^{\text{2}}\\ & \text{= }1.96^{\text{2}}\times 0.862\times 0.138/\text{0}{\text{.05}}^{\text{2}}\\ & \text{= }183 \end{array}$$

where,

n = minimum required sample size
Z = 1.96 at 95% Confidence Interval
p = prevalence of periodontal disease, 86.27%^8^
e = margin of error, 5%.

The data were recorded on the proforma based on World Health Organization (WHO) Oral Health Assessment Survey Form.^9^ After a detailed questionnaire, oral hygiene status was assessed using Simplified Oral Hygiene Index (OHI-S) followed by periodontal status using the Community Periodontal Index (CPI).^9,10^ The dentition was divided into six sextants. Each sextant was assigned a code number and the condition of the worst affected site in that sextant was recorded. The highest CPI and loss of attachment (LOA) scores of any sextant were recorded as the respective CPI and LOA for that individual. The examination was done using mouth mirror, No. 23 explorer (Shepherd's hook) and CPI-C probe. The overall OHI-S was obtained by adding the two components Simplified Debris Index (DI-S) and the Simplified Calculus Index (CI-S). OHI-S score was interpreted as: Good (0.0-1.2), Fair (1.3-3.0) and Poor (3.1-6.0).

The BMI values were calculated using standard formula (weight in kilograms divided by the square of height in meters). BMI was categorised as underweight (<18.5), normal weight (18.5-24.9), overweight (25.0-29.9) and obese (≥30).^11^ Smoking behaviour was categorised as current smokers (smoked ≥100 cigarettes in their lifetime and currently smoke), former smokers (smoked ≥100 cigarettes in their lifetime and do not currently smoke) and Non-smokers (not smoked ≥100 cigarettes in their lifetime and do not currently smoke).^12^

Data were entered in Statistical Package for Social Sciences version 23 and analysed. The descriptive statistics were presented as frequency, percentage, mean, standard deviation and standard errors of mean.

## RESULTS

Regarding the overall periodontal status of the 183 participants, 104 (56.83%) had CPI score of 2 and 84 (45.90%) had LOA score of 1 (Table 1). Healthy periodontal condition CPI 0 was found only in 2 (1.09%) and LOA 0 in 79 (43.17%) of the examined individuals indicating the prevalence of gingival disease (corresponding to CPI 1 and 2) as 136 (74.32%) and the prevalence of periodontal disease (corresponding to LOA 1, 2, 3 and 4) in 104 (56.83%) participants (Table 1). The mean values of CPI and LOA scores per sextant with the different age categories in both the sexes are presented (Table 2 and Table 3).

**Table 1:** The community periodontal index scores.

CPI score	n (%)
CPI 0 (healthy)	2 (1.09)
CPI 1 (bleeding)	32 (17.49)
CPI 2 (calculus)	104 (56.83)
CPI 3 (pocket 4-5mm)	44 (24.04)
CPI 4 (pocket ≥ 6mm)	1 (0.55)
CPI X (excluded, < 2 teeth present)	-
LOA score	
LOA 0 (0-3mm)	79 (43.17)
LOA 1 (4-5mm)	84 (45.90)
LOA 2 (6-8mm)	19 (10.38)
LOA 3 (9-11mm)	1 (0.55)
LOA 4 (≥ 12mm)	-
LOA X (excluded)	-

**Table 2:** CPI scores affected in each sextant according to age and sex.

CPI score per sextant	Age group (years)	Sex	Mean±SD	SEM
	≤ 35	Female	1.32±0.91	0.120
		Male	1.81±0.66	0.101
	36 to 55	Female	1.93±0.83	0.159
17/16		Males	2.09±0.58	0.101
	≥ 56	Female	2.00±0.54	0.189
		Male	2.47±0.99	0.256
	≤ 35	Female	0.61±0.65	0.086
		Male	0.93±0.67	0.102
	36 to 55	Female	1.11±0.93	0.180
11		Male	1.30±0.81	0.141
	≥ 56	Female	1.50±0.76	0.267
		Male	1.47±1.19	0.307
	≤ 35	Female	1.26±0.89	0.119
		Male	1.77±0.89	0.137
	36 to 55	Female	1.78±0.93	0.180
26/27		Male	1.97±0.74	0.131
	≥ 56	Female	2.00±0.54	0.189
		Male	2.50±0.86	0.228
	≤ 35	Female	1.32±0.85	0.112
		Male	1.56±0.88	0.134
	36 to 55	Female	1.52±1.05	0.202
36/37		Male	1.91±0.88	0.153
	≥ 56	Female	2.00±0.54	0.189
		Male	2.270±1.16	0.300
	≤ 35	Female	0.81±0.77	0.101
		Male	1.19±0.79	0.121
	36 to 55	Female	1.19±0.89	0.176
31		Male	1.39±0.93	0.162
	≥ 56	Female	1.57±0.79	0.297
		Male	2.00±1.19	0.309
46/47	≤ 35	Female	1.41±0.85	0.113
		Male	1.84±0.69	0.105
	36 to 55	Female	1.67±0.92	0.177
		Male	2.09±0.68	0.118
	≥ 56	Female	2.00±0.54	0.189
		Male	2.29±1.14	0.304

**Table 3:** Loss of attachment score in each sextant according to age and sex.

LOA score per sextant	Age group (years)	Sex	Mean±SD	SEM
	≤ 35	Female	0.18±0.38	0.051
		Male	0.35±0.48	0.074
	36 to 55	Female	0.52±0.58	0.112
		Male	0.73±0.67	0.117
	≥ 56	Female	1.13±0.35	0.125
17/16		Male	1.13±0.83	0.215
	≤ 35	Female	0.04±0.19	0.025
		Male	0.02±0.15	0.023
	36 to 55	Female	-	-
11		Male	0.21±0.49	0.084
	≥ 56	Female	0.50±0.76	0.267
		Male	0.33±0.49	0.126
	≤ 35	Female	0.14±0.35	0.046
		Male	0.21±0.41	0.063
	36 to 55	Female	0.41±0.57	0.110
26/27		Male	0.61±0.76	0.137
	≥ 56	Female	0.63±0.52	0.183
		Male	1.21±0.80	0.214
	≤ 35	Female	0.19±0.44	0.058
		Male	0.30±0.47	0.071
	36 to 55	Female	0.33±0.48	0.092
36/37		Male	0.39±0.61	0.106
	≥ 56	Female	0.63±0.52	0.183
		Male	1.00±0.85	0.218
	≤ 35	Female	0.11±0.41	0.054
		Male	0.12±0.39	0.060
	36 to 55	Female	0.08±0.27	0.053
31		Male	0.33±0.54	0.094
	≥ 56	Female	0.57±0.79	0.297
		Male	0.40±0.51	0.131
		
	≤ 35	Female	0.11±0.31	0.041
		Male	0.16±0.37	0.057
	36 to 55	Female	0.22±0.42	0.082
46/47		Male	0.58±0.67	0.115
	≥ 56	Female	0.88±0.35	0.125
		Male	0.86±0.77	0.206

Oral hygiene status as assessed by OHI-S was found to be “fair” in majority 114 (62.30%) of the individuals (Table 4). The mean OHI-S score of the participants was 1.67±0.89 (Table 5) with mean DI-S score of 0.99±0.42 (SEM=0.03) and mean CI-S score of 0.68±0.56 (SEM = 0.04).

**Table 4:** Oral hygiene status, practices, and demographics.

	Female n (%)	Male n (%)	Total n (%)
**Simplified Oral Hygiene Index (OHI-S)**			
Good	35 (19.13)	18 (9.83)	53 (28.96)
Fair	53 (28.96)	61 (33.34)	114 (62.30)
Poor	4 (2.18)	12 (6.56)	16 (8.74)
**Dental visits**			
First visit	34 (18.58)	46 (25.14)	80 (43.72)
Frequently	23 (12.57)	8 (4.37)	31 (16.94)
Occasionally	35 (19.13)	37 (20.22)	72 (39.34)
**Tooth cleaning device**			
Toothbrush	92 (50.27)	91 (49.73)	183 (100)
**Interdental aids usage**			
Dental floss	3 (1.64)	-	3 (1.64)
No aids	89 (48.63)	91 (49.73)	180 (98.36)
**Mouthwash usage**			
Yes	4 (2.18)	-	4 (2.18)
No	88 (48.09)	91 (49.73)	179 (97.82)
**Type of brush**			
Hard	3 (1.64)	6 (3.28)	9 (4.92)
Medium	41 (22.40)	65 (35.52)	106 (57.92)
Soft	48 (26.23)	20 (10.93)	68 (37.16)
**Dentifrice type**			
Fluoridated	90 (49.18)	90 (49.18)	180 (98.36)
Non-fluoridated	2 (1.09)	1 (0.55)	3 (1.64)
**Brushing methods**			
Scrub (horizontal)	90 (49.18)	90 (49.18)	180 (98.36)
Circular	2 (1.09)	1 (0.55)	3 (1.64)
**Brushing frequency**			
Once per day	56 (30.60)	71 (38.80)	127 (69.40)
Twice daily	35 (19.13)	18 (9.83)	53 (28.96)
Occasionally	1 (0.55)	2 (1.09)	3 (1.64)
**Smoking habits**			
Current smoker	3 (1.64)	32 (17.49)	35 (19.13)
Former smoker	2 (1.09)	9 (4.92)	11 (6.01)
Non-smoker	87 (47.54)	50 (27.32)	137 (74.86)
**Form of smoking**			
Cigarettes	5 (2.73)	41 (22.40)	46 (25.14)
**Education**			
Illiterate	7 (3.83)	5 (2.73)	12 (6.56)
Primary and upper primary (grade 1-8)	1 (0.55)	5 (2.73)	6 (3.28)
Secondary and higher secondary (grade 9-12)	54 (29.51)	32 (17.49)	86 (46.99)
Bachelors and above	30 (16.39)	49 (26.78)	79 (43.17)
**Occupation**			
Army	-	3 (1.64)	3 (1.64)
Business	4 (2.18)	15 (8.20)	19 (10.38)
Creative and sports	3 (1.64)	2 (1.09)	5 (2.73)
Health and social worker	9 (4.92)	4 (2.18)	13 (7.10)
Hospitality	-	4 (2.18)	4 (2.18)
Manual worker	-	13 (7.10)	13 (7.10)
Service (govt. and private, white collar)	6 (3.28)	26 (14.21)	32 (17.49)
Student	31 (16.94)	17 (9.29)	48 (26.23)
Unemployed	39 (21.31)	7 (3.83)	46 (25.14)
Total	92 (50.27)	91 (49.73)	183 (100)

**Table 5:** Periodontal and oral hygiene status in different sex and age groups.

Age (years)	n (%)	Sex	n (%)	CPI		LOA		OHI-S		BMI	
				Mean±SD	SEM	Mean±SD	SEM	Mean±SD	SEM	Mean±SD	SEM
≤ 35	100 (54.64)	Female	57 (31.15)	1.74±0.75	0.09	0.35±0.58	0.07	1.23±0.77	0.10	21.17±2.97	0.39
		Male	43 (23.50)	2.12±0.59	0.08	0.56±0.55	0.08	1.62±0.81	0.12	23.34±2.70	0.41
36 to 55	60 (32.79)	Female	27 (14.75)	2.15±0.53	0.10	0.67±0.62	0.11	1.82±0.66	0.12	25.55±3.39	0.65
Male	33 (18.03)	2.21±0.65	0.11	0.94±0.61	0.10	2.02±0.98	0.17	24.95±1.94	0.33
≥ 56	23 (12.57)	Female	8 (4.37)	2.00±0.54	0.19	1.25±0.46	0.16	2.07±0.51	0.18	26.45±4.55	1.60
		Male	15 (8.20)	2.60±0.74	0.19	1.47±0.74	0.19	2.30±1.10	0.28	25.36±4.41	1.13
		Female	92 (50.27)	1.88±0.69	0.07	0.52±0.64	0.06	1.47±0.78	0.81	22.92±3.92	0.40
		Male	91 (49.73)	2.23±0.65	0.06	0.85±0.68	0.07	1.88±0.95	0.09	24.25±2.92	0.30
		Total	183 (100)	2.05±0.69	0.05	0.68±0.68	0.05	1.67±0.89	0.06	23.58±3.52	0.25

SD = standard deviation; SEM = standard error of mean.

For most participants, it was their first dental visit (80, 43.72%). All of the examined individuals 183 (100%) used toothbrush exclusively to clean their teeth, but only 3 (1.64%) and 4 (2.18%) participants (female individuals in both categories) used interdental aids (dental floss) and mouthwashes respectively (Table 4). Most of them brushed their teeth once a day (127, 69.40%) using medium-bristled toothbrushes (106, 57.92%) and fluoridated toothpaste 180 (98.36%).

Among 183 individuals, 92 (50.27%) were females (Table 4, 5). Similar to the pattern of Grewal et al.^13^ all the participants were categorised into three age groups namely ≤ 35 years, 36 to 55 years, ≥ 56 years (Table 5). Age categories were found to be associated with CPI, LOA, OHI-S and BMI. In ≤ 35 years, mean scores of CPI was 1.90±0.70, LOA was 0.44±0.57, OHI-S was 1.39±0.81 and BMI was 22.11±3.04. In 36 to 55 years individuals, the mean values were: CPI = 2.18±0.59, LOA = 0.82±0.62, OHI-S = 1.93±0.85 and BMI = 25.22 ± 2.69. In participants of ≥ 56 years, the mean score of CPI was 2.39 ± 0.72, LOA was 1.39±0.66, OHI-S was 2.22±0.93 and BMI was 25.74±4.39. The overall mean scores as well as in individual categories are stated in (Table 5). With increasing age, the periodontal status (CPI and LOA scores), oral hygiene status (OHI-S score) and BMI mean values deteriorated, in both the sexes.

The mean BMI score was 23.58±3.52 (SEM = 0.26; minimum = 16.66; maximum = 38.27) with 113 (61.75%) participants of normal weight (18.5 to 24.9) forming the majority, while 55 (30.05%) were overweight, 8 (4.37%) underweight and 7 (3.83%) obese. When the BMI scores and periodontal status were compared, the mean values of BMI were seen to increase with increasing CPI, LOA and OHI-S scores.

Majority of the study participants (137, 79.4%) were non-smokers (Table 2). The form of smoking among smokers was found to be exclusively cigarettes. When smoking status was compared with CPI, LOA and OHI-S scores, periodontal and oral hygiene status was seen to be better in non-smokers (CPI = 1.97±0.69; LOA = 0.55±0.62; OHI-S = 1.52±0.84) , but not much difference was appreciated among current (CPI = 2.20±0.58; LOA = 1.03±0.71; OHI-S = 2.12±1.00) and former smokers (CPI = 2.64±0.67; LOA = 1.27±0.65; OHI-S = 2.14±0.36). Smoking status and sex also showed positive relation with more male smokers in both current as well as former smoker categories (Table 2).

The mean age of the population was 36.37±14.43 years (SEM = 1.067, minimum 16 years, maximum 72 years) of which most were either students 48 (26.23%) or unemployed 46 (25.14%). The education level “secondary and higher secondary” category had majority 86 (46.99%) of the participants followed by “bachelors and above” category (Table 2). Of all participants, 34 (18.58%) suffered from cardiac diseases, 16 (8.74%) had diabetes, 1 (0.54%) had gastrointestinal disorder and 3 (1.63%) had thyroid disorders. In total, 133 (72.68%) reported no underlying systemic condition.

## DISCUSSION

Oral diseases mainly the periodontal disease and dental caries are one of the commonest chronic diseases affecting mankind. In the past, diseases of the oral cavity have been viewed separately from those of the rest of the body. However, in recent years, efforts have been made to recognise oral health as an integral component of general health. Healthy teeth along with its supporting structure, the periodontium is vital for the maintenance of teeth in oral cavity and thus important for oral health. The initial stage gingivitis progresses in a sequential manner towards periodontitis ultimately resulting in tooth loss. Thus, if oral health diseases can be identified at an early stage, tooth loss can be prevented. The causes for tooth loss: periodontal disease and dental caries, both are entirely preventable by regular oral hygiene maintenance.

Though it is well-known and often advocated that practising just simple measures of oral hygiene like brushing twice daily with soft-bristle toothbrush, fluoridated toothpaste, using interdental aids, and visiting the dentist twice a year for regular check-up and oral prophylactic treatment can reduce tooth loss drastically, individuals seldom practise so. The present study provides information on oral hygiene status, oral hygiene practices and periodontal health of 183 people of age above 15 years visiting a tertiary care dental hospital.

Oral hygiene status was found to be “fair” in most of the population 114 (62.30%). This was similar to study by Bhattarai et al.,^14^ Umoh and Azodo^6^ and Olabisi et al.^15^ The high values of the oral hygiene index and its components (debris and calculus indices) suggest a neglect of tooth cleaning and oral health awareness. It is further supported by the frequency of dental visits by the population. For most of the sample population it was their first dental visit 80 (43.72%). This is in contrast to Bhattarai et al. who reported 65.5% had visited a dentist before.^14^ The frequency of brushing was reported “once daily” by most. This is similar to findings of Baishya et al.^8^ Asgari et al.^16^ El Bcheraoui et al.^17^ and Shah and ElHaddad.^18^ Majority did not use dental floss or other interdental aids, similar to the same studies.^16-18^ Despite advocacy by dentists and related organisations to brush twice daily and use interdental aids, the individuals do not seem motivated enough to practice that. The tooth cleaning devices were exclusively toothbrushes and toothpastes where 180 (98.36%) of the subjects used fluoridated toothpaste. This is in contrast with the study of Pradhan et al.^5^ where the sample population used several other tooth cleaning methods including finger, twig, tooth powder, charcoal, ash, sand and tobacco but similar to Bhattarai et al.^14^ The sample population in Pradhan et al. was rural whereas Bhattarai et al. was urban like present study. Though scrub (horizontal) brushing technique is not recommended by dentists, it was the technique preferred by the participants 180 (98.4%). It could be due to convenience and lack of knowledge regarding better brushing techniques like modified Bass, modified Stillman, etc.

In the present study, healthy periodontal condition CPI 0 was found only in 2 (1.09%) and LOA 0 in 79 (43.17%) of the examined individuals indicating the presence of gingival disease in 136 (74.32%) and periodontal disease in 104 (56.83%) participants. This is in consonance with other studies.^4,5,19-21^ Calculus (Code 2) and shallow pocket (Code 3) were the most frequently observed conditions followed by bleeding on probing (Code 1).^6,13,19-23^ Lack of oral health awareness and negligence from people could be the factors responsible for this even though the study was conducted in urban population.

The number of healthy sextants decreased with advancing age which is in accordance with available literature reporting increasing age with increasing severity of periodontitis.^2,3,7,8,21,22,24^ About “Education”, majority of the sample were secondary and higher secondary above. One reason could be because of lack of oral health awareness in less educated individuals, they don't go to dental hospital unless they have to. Some epidemiological studies have shown positive association between BMI and periodontitis while others have not.^24^ The current study also showed higher BMI scores with worsening oral and periodontal status.

Though, periodontal status and oral hygiene status were observed to be better in non-smokers, similar to other studies, most 137 (79.4%) of the study participants were non-smokers.^2,17,18,24^ Study with equally distributed participants would show more accurate finding. As smoking is considered a taboo or adverse habit in a society like ours, the participants may not have reported the true smoking status.^24^ Female participants showed better oral hygiene practices and better oral and periodontal health. This is similar to other studies within Nepal and abroad.^2,7,15,16,19,21^ This could be because of female individuals being more concerned regarding oral hygiene and visit dentist more often. Male also have other deleterious habits like smoking cigarette, chewing pan/tobacco, taking gutkha, that negatively affect oral health. Smoking status and sex also showed positive association with more male smokers both current as well as former similar to Gupta et al.^2^ Dahal et al.^7^ and Kundu et al.^21^ The result may not be accurate because due to social disparities, the female smokers may not have reported truthfully.

The present study showed a high prevalence of gingival disease, calculus and shallow pockets in the examined population which indicate that the oral hygiene methods employed and the frequency of dental visits by the population are not adequate or effective enough. As the updated information on oral and periodontal health of Nepali adults is limited, this assessment would be helpful to national policy makers on oral health and intervention measures to prevent periodontal diseases and develop future programs to improve oral health.

The limitations are: since the CPI and OHI-S measurements were done only in the index teeth, it can overestimate or underestimate the prevalence of periodontal disease and oral hygiene status respectively. Similarly, the relation between method of cleaning teeth, pack years of smoking, oral hygiene status, and periodontal disease were not considered. Other limitations include: it was a single-centre observational study with small sample size and short duration. Hence multi-centre studies with larger sample size would give more accurate representation of periodontal status and factors affecting the periodontal health in Nepalese population.

## CONCLUSIONS

In current study, more than half of the participants had periodontal disease. The findings also suggest a positive relation of deteriorating oral and periodontal health status with age, sex, oral hygiene practice, poor oral habits (like smoking), and BMI. A very low priority is placed upon its prevention and treatment due to relatively low mortality and morbidity compared to other diseases and other factors like knowledge, cost, availability, and accessibility of services. Though, there is an upward trend in attending clinics and hospital facilities in urban areas, the result shows that there is a need to give more attention to periodontal disease. Emphasis on brushing twice daily with soft-bristled toothbrush and fluoridated toothpaste, usage of interdental aids and visiting a dentist twice a year is still lacking.

In order to achieve ideal oral health status and periodontal health, appropriate preventive and periodic periodontal therapies should be provided. Also, to bring down the prevalence of periodontal diseases to decrease the tooth loss and improve the oral health related quality of life, special emphasis should be given on oral health education and motivation both from government as well as private sectors.
